# The Future of Transportation: Ethical, Legal, Social and Economic Impacts of Self-driving Vehicles in the Year 2025

**DOI:** 10.1007/s11948-019-00130-2

**Published:** 2019-09-03

**Authors:** Mark Ryan

**Affiliations:** University of Twente, Enschede, The Netherlands

**Keywords:** Ethics of self-driving vehicles, Self-driving cars, Artificial intelligence, Big data, Philosophy of technology, Scenario foresight analysis

## Abstract

Self-driving vehicles (SDVs) offer great potential to improve efficiency on roads, reduce traffic accidents, increase productivity, and minimise our environmental impact in the process. However, they have also seen resistance from different groups claiming that they are unsafe, pose a risk of being hacked, will threaten jobs, and increase environmental pollution from increased driving as a result of their convenience. In order to reap the benefits of SDVs, while avoiding some of the many pitfalls, it is important to effectively determine what challenges we will face in the future and what steps need to be taken now to avoid them. The approach taken in this paper is the construction of a likely future (the year 2025), through the process of a policy scenario methodology, if we continue certain trajectories over the coming years. The purpose of this is to articulate issues we currently face and the construction of a foresight analysis of how these may develop in the next 6 years. It will highlight many of the key facilitators and inhibitors behind this change and the societal impacts caused as a result. This paper will synthesise the wide range of ethical, legal, social and economic impacts that may result from SDV use and implementation by 2025, such as issues of autonomy, privacy, liability, security, data protection, and safety. It will conclude with providing steps that we need to take to avoid these pitfalls, while ensuring we reap the benefits that SDVs bring.

## Introduction

Rarely will a day go by where we do not see a new story, controversy, or debate surrounding self-driving vehicles (SDVs). Despite the repeated influx of information on SDVs, such prevalent discussion should not reduce the importance of continued evaluation of the potential issues this emerging technology will bring. While there has been an abundance of ethical, legal, and social research done already (De Sio 2017; Nyholm 2018; Nyholm and Smids 2016), the range and diversity of concerns are rarely brought together in a single body of work, nor does prior research take into account specific and projected timelines for issues which may emerge from the new technology, with relevant policy responses to those issues. This paper is the first to provide such a comprehensive approach through the use of a ‘policy scenario’ methodology, providing a stakeholder-engagement-centred approach, to examine the impacts of SDVs by the year 2025.

Debate about the societal implications of SDVs often become pre-empted by discussions about widespread level 5 automation, where the vehicle performs all driving functions in all circumstances, which is not expected for several decades, so there tends to be less urgency to consider more immediate impacts. There is a need, then, to consider nearer-term developments in the use of SDVs to effectively deal with issues that we face in the next five to 6 years. One possible approach for meeting this need to consider these more immediate concerns is the use of scenarios. A scenario is not a future reality, but is a plausible future reality, grounded on current understandings of technological developments and issues surrounding those technologies (Durance and Godet 2010). The purpose of a scenario in this instance is to provide a well-formulated, and plausible, prediction of the state of SDVs in the year 2025 and potential impacts that they may cause. Therefore, the scenario presented in this paper is grounded on the views of experts in the field (generated in a 1-day workshop), stakeholder engagement (generated through outreach for iterations on the scenario), as well as integration of current scientific research in the field (literature analysis).

## Methodology

This paper reports on work from an existing project, the SHERPA Project,^1^ convened to evaluate five applications of emerging technologies and their potential societal implications by 2025; one of those technologies was SDVs (full outline can be found at Wright et al. 2019). The project adopted a scenario methodology to assist in the identification of ethical, legal, social and economic issues related to self-driving vehicles in the coming 6 years. Scenarios are “a tool for ordering one’s perceptions about alternative future environments in which one’s decisions might be played out concretely, so people can help people make better decisions” (Schwartz 1998, p. 4). While there are many benefits of using scenarios, there are also several drawbacks. For example, scenario approaches are often unstructured, making it difficult to find clear-cut guidance about the issues at stake. Some scenarios are very interesting thought-experiments but provide little guidance on what actions should be taken in practice. Particularly, when they need to be understood by policymakers, vague and convoluted scenarios that require elaborate deconstruction reduce their effectiveness.

From the six scenario approaches (see Table 1) outlined in the SHERPA project, we used the policy scenario for the SDV case, as that scenario construction is the most coherent approach for policymakers. The aim is to have one concise, understandable, and plausible scenario to show policymakers what the most pressing issues and impacts are and steps to achieve a desirable future for SDV use. Policy scenarios are intended to provide an effective approach to identify a diversity of issues, but constructed in an articulate and precise way to support policymakers in the following ways: “To explore possible consequences of current trends; to engage stakeholders; to uncover issues that might otherwise be overlooked; to help decision-making; to consider desired and undesired futures; to determine what steps should be taken to reach the desired future and avoid an undesired future” (Wright et al. 2019, p. 5).

**Table 1:** See Wright et al. (2019)

Scenario type	Description
‘Best-case, status quo, worst-case’	This tripartite scenario creates three future scenarios: a best-case; one if we continue current trends; and a worst-case. This may be confusing or misleading for policymakers, as it gives three contradictory potential futures, making it challenging to pinpoint what type of policy is required
Orthogonal futures	This scenario is grounded on a four-quadrant matrix of possible futures (X and Y axis), which represent polar issues to be discussed. It may be too simplistic and overlooks many of the rich nuances required for policy implementation
Dark scenario	Dark scenarios focus on the worst-case possible future. It simply tells policymakers what to avoid, and not how to reach a desirable future
Ethical dilemma scenario	Commonly used in philosophical discourse or thought experiments to identify an issue, but often there is no clear-cut course of action to take
Narrative scenario	This approach tells a scenario in a story-like context. While stories are good to allow reader engagement, they often do not allow for a comprehensive evaluation of the diversity of issues relating to emerging technologies
Policy scenario	This approach incorporates a diversity of stakeholders to illustrate a scenario. It is based on plausible impacts and issues and provides a clear outline for policymakers to ensure a desirable future and avoid undesirable impacts

The policy scenario aims to establish plausible outcomes, which are evidenced and grounded on current scientific understanding and projections. Scenarios are a very effective tool for policymakers, if they contain a strong degree of plausibility (Volkery and Ribeiro 2009). A scenario should indicate a future timeline that has a level of predictability and does not veer too close to science fiction (Cairns and Wright 2018, pp. 34–45). Therefore, this scenario opts for near-term developments in SDVs, i.e. within the next 5–6 years. This timeline also creates an urgency for policymakers to act—an important component of the policy scenario methodology. Stakeholder engagement is a key component of scenario development and has been integrated in this methodology (Duckett et al. 2017). Stakeholders have been engaged from the scenario’s conception, right through to its final construction. One of the key factors for policymakers is to have stakeholder engagement and feedback, which this scenario development process fulfils. This lends strength to the findings and recommendations for decisions on SDVs policy implementation and greater credence to its scientific plausibility and probability.

As detailed in the SHERPA report, the SDV scenario went through four iterative stages, beginning with a 1-day workshop, consisting of 20 experts. A single, or multiple, participant(s) from the project was responsible for the resultant iterations of the scenario. The participants came from a wide range of different backgrounds, experiences, and disciplines, such as standardisation bodies, SDV testing, computer scientists, engineers, psychologists, AI specialists, cybersecurity experts, ethicists, and legal scholars. The SDV workshop was split into several sections that would mirror the sections of the scenario’s construction: SDV technological development in 2025^2^; driving forces and barriers for SDVs; the ethical, legal, social, and economic impacts of SDVs; how to mitigate the negative and accentuate the positive impacts of SDVs; and how to reach a desirable future. These sections were split between group-work, open discussion, and critical dialogue of SDVs, so it often involved collective brainstorming and evaluations of the topics discussed.^3^

Following the workshop, a draft scenario was sent back to the workshop participants for their input and feedback. Further insights were retrieved from relevant journal publications and articles on SDVs to incorporate any additional issues overlooked, while also supporting the scientific validity of the views purported in the scenario construction. Additional literature was used to support the scientific merit of the scenario, to show that many of the topics being discussed are legitimate current concerns.^4^ A revised scenario was sent to a wider range of experts (30+ people) for the third iteration; before finally posting it on a public platform to receive public engagement (100+ people). The aim of this process was to create a nuanced, cohesive, and consensus-driven scenario for SDV development in the coming 6 years. Overall, the scenario incorporated feedback until the scenario was at a stable state for dissemination on the SHERPA website. This paper is a revised and more detailed examination of the topics outlined in the report for this project. However, there are sections that are quite similar to the original version, but for stylistic and cogency reasons, there will be a strong degree of overlap with the project report (Table 2).

**Table 2:** Six levels of automation (NHTSA 2017)

The six stages towards full automobile automation (NHTSA 2017)
Level 0 refers to automobiles that have no automation whatsoever, whereby the driver performs all actions and driving tasks
Level 1 refers to the driver assistance stage, whereby the vehicle is still controlled by the drive, but there are some features to assist the individual in their driving
Level 2 refers to partial automation, where there is driving automation in certain aspects of the driving experience, i.e., acceleration and steering. However, the driver needs to remain fully engaged throughout and take over if necessary
Level 3 refers to ‘conditional automation’, where more control is given to the vehicle, particularly environmental monitoring, but the driver must be ready to take over if required
Level 4 depicts high automation of the vehicle. The vehicle has the capacity to respond to most aspects of the driving experience, leaving almost full disengagement of the driver
Level 5 the vehicle is ‘capable of performing all driving functions under all conditions’ (NHTSA 2017)

## Scenario of Self-Driving Vehicles Between 2019 and 2025 (Ryan 2019)

In 2025, SDVs are used in different urban areas throughout the world. 38-year-old Software Developer Hans Adrian uses his self-driving car to go to his office in München every morning, which was one of the first places to roll them out. “So far, so good”, explains Hans, who has been using his SDV for over 4 months now. “I am able to work in my car while commuting. When you factor in an hour commute each way, I get back 10 h of my life that is lost in the commute every week. I sit back with my laptop, while listening to Spotify. It’s great!” Hans’ Waymo Centauri b is one of the few permitted self-driving car models on the market and has been one of the most widely adopted of these vehicles, so far.^5^ The Centauri b is still in the hybridisation stage towards full automation, having both automated, semi-automated, and manual driving possibilities at level 4 automation. Legally, Hans can only drive fully automated within designated areas of München, but for most other places the car must be in semi-automated or manual mode. “It is a nuisance when I have to drive outside München. It takes a while to get used to the wheel again. But I understand that it will take other cities time before they catch up with us,” Hans claims, as the vehicle navigates through his neighbourhood in level 4 automation. His car changes lanes and stops at pedestrian lights, gives way at roundabouts, while allowing him the comfort to catch up on work or just relax and take in the scenery.

So far, SDVs have gained universal integration in only seven cities in the world, but there are hopes that this number will increase dramatically by 2030. Many of the leading car manufacturers and experts estimate that this number will be between 50 and 70 cities by the end of the decade. Some of the most pioneering and revolutionary developments have been coming from Silicon Valley, while the most prolific countries behind SDV development have been the US, South Korea, the UK, Japan, China, and Germany. The US has been the real innovator behind SDVs, with more than 40 cities piloting SDVs as far back as 2017, dwarfing all other countries in comparison (Hao 2017). At the start of 2025, there were 100 cities in the US piloting SDVs, and this number is set to increase dramatically by 2030.

Hans has reaped the benefits of autonomous driving, but only after he passed his SDV driving test. This test consists of both an SDV-driving theory test on how to operate and use the vehicle, with a strong emphasis on shifting from driving to automation, as well as a practical driving examination. Hans was required by German law to undertake 12 driving lessons prior to taking in-car tests. In addition, cities integrating SDVs must also be authorised with the National Self-Driving Vehicle Transportation Board (NSDVTB) and the vehicle owner must be registered with the Department of Self-Driving Vehicles Authority (DSDVA).

The vehicle itself must pass strict manufacturing standards before being allowed on the market. Outside of these designated areas, cars must function at level 3 capacity—limited automation. The car senses when conditions require the driver to retake control and provides enough transition time for the driver to do so. Some SDV companies wanted to skip this stage, but the limitations of technological organisation, the interaction with manual drivers, and the lack of infrastructure to accommodate this move have been too problematic. In areas where there are mixed drivers (automation and non-automation), SDVs must have a level 3 option for legal reasons. One of the main reasons behind these laws is to ensure safety, which has been one of the main facilitators of the development of SDVs in the first place.^6^

## Facilitators of SDVs Between 2019 and 2025

*Safety facilitators* Approximately 90% of crashes are the result of mistakes by the driver and while road deaths have been decreasing, they were as high as 1.4 million in 2015 (NHTSA 2013, 2017; WHO 2018). Over the past 10 years, safety has been one of the strongest motivators among the driving industry and road safety organisations for the implementation of SDVs but we have yet to reap their true benefits because of SDVs’ low level of use. The National Safety Council’s ‘Road to Zero’ campaign is an ambitious goal to have zero automobile-related deaths in the United States by 2050, which may be feasible if they are successfully adopted nationally (Ecola et al. 2018). As far back as 2017, there have been studies to show that deploying SDVs when they are only marginally safer (say, 10%) than human drivers, would still have a dramatic impact on reducing road deaths. Policymakers around the world have largely indicated that waiting for SDVs to be far safer (say, 75–95%) than human drivers is not an option because of how long it would take to reach that stage (Kalra and Groves 2017).

*Social facilitators* The general public are enthused by the fact that SDVs may offer people the ability to work, sleep, read, eat, or watch TV, while “driving”. In 2014, the American Trucking Association (ATA) predicted that there would be a huge shortage of truck drivers, which would necessitate the development of self-driving trucks. Their prediction of 175,000 drivers by 2024 came up short of the reported 215,000-figure taken in November 2024 (Seattle Truck Law PLLC 2018).

*Environmental facilitators* In the cities where SDVs have been integrated, there are positive indications of carbon emission reductions. Many environmental agencies have demanded more environmentally-sustainable vehicles since the Kyoto and Paris climate agreements, and cities view electric SDVs as one way to meet their EU carbon emission requirements (European Environment Agency 2016). Since 2023, several auto manufacturers have been testing single-user SDVs to bring people from their homes to public SDV buses, which would further reduce environmental impact, while reducing costs.

*Economic facilitators* While the price of SDVs has been decreasing every year, they are still more expensive than non-automated cars. There has been a recent influx of SDV car-sharing and ride-sharing apps to reduce costs, so that the cars do not sit idle in people’s garages or parking lots and can be used throughout the day (Ohnsman 2018). Fuel costs are lower because of greater fuel-efficiency and when they reach widespread level 4 integration, and safety is improved, production costs will decrease because there will be no need for airbags and steering wheels (Davies 2018). Between the 2020 and 2025 periods, many new non-traditional players, such as ICT and data analytics companies, have emerged in the SDV automotive market. Some automotive companies view SDVs as a threat because they cannot put the same kind of investments into developing these technologies as much as their larger automotive counterparts. Fiat, who had been struggling for several years, has closed several of their manufacturing depots, claiming that the shift to automation has massively impacted their sales (Eisenstein 2019).

*Market facilitators* Over the years, many social critics have stated that the SDV market is supply-driven (McCarthy 2018). SDV manufacturers have seen the benefit of SDVs for goods transportation and data analytics (DHL 2014; Hawthorne-Castro 2018). Auto manufacturers have been hugely competitive in the race to develop SDVs, bringing global success and prestige to their companies. Companies have been extensively patenting their cars, products, and services to lock customers into their brand. However, the notion of automotive branding has been changing over the past few years, with a shift from luxury, status and appearance, towards efficiency, safety, and functionality.

*Efficiency and productivity facilitators* As a result of greater driving efficiency, Traffic Management Authorities have heralded SDVs for their ability to reduce traffic jams and congestion, identifying better routes to take, more sustainable driving, and a reduction in crashes holding up traffic flow. Despite SDVs being heralded as a way for people to get extra sleeping or relaxation time on their commutes to work, some businesses view them as offering the possibility of cutting out needless ‘driving time’, allowing staff to work while in the vehicle.

*Political facilitators* National Ministers for Transportation are encouraging the uptake of SDVs because of their potential to reduce lane size and quantity of lanes due to driving efficiency. However, this has proven difficult in many larger US cities because of the sheer difficulty of implementation. SDVs also promise to reduce road deaths, which will reduce costs on governmental healthcare spending. Furthermore, people will be able to commute longer distances because of the comfort of SDVs, thus moving further from work, which will reduce the strain and congestion in many urban areas.

## Potential Barriers and Inhibitors for SDVs Between 2019 and 2025

*Safety and security barriers* Many different safety issues have slowed down the development of SDVs. The safety of automated vehicles has been a primary concern amongst road-users and pedestrians, especially following some of the highly-publicised deaths, such as the Tesla Model S in 2016 (Stilgoe 2018). People have found it difficult to put their safety in the hands of an autonomous machine for fear of technical or systems failures and malfunctions. There has also been a concern raised about pedestrian safety and algorithmic bias, following the early discovery that SDVs image-recognition held a bias towards pedestrians with darker skin colour (Cuthbertson 2019). An emphasis was placed on research and testing to ensure that skin colour biases have been practically eliminated in the past 6 years.

While crashes with SDVs have decreased over the past few years, they are still more risk-prone in terms of accidents per mile driven than driver-controlled vehicles. Even going back as far as 2017, figures indicated accident rates for every 48,000 miles driven for SDVs, compared to every 2.08 million miles driven for non-autonomous cars (Johnsen et al. 2017, p. 33). Mode transitions has raised additional safety issues, such as distraction, loss of situational awareness, and high workload during take-over. These factors have proven to be inhibitors to the successful development of SDVs and are issues that are constantly being tested and rectified. Many people have also been worried about the security risk of SDVs, such as hacking, manipulation and malicious activity.

*Technical barriers* There have been many technological barriers to SDV development, including issues around hacking, data and vehicle security. Initially, steering systems had built-in processes to determine abnormal instructions, but after a few minor concerns relating to compromised commands, SDVs were implemented with an emergency procedure that would override individual tasks and take control and bring vehicle to a safe stopping position in the case of suspicious activity. Auto manufacturers have been pressured to provide increased AI transparency, which has inhibited the speed of development, as have the challenges of ensuring adequate software and hardware updates. Locations need to have 5G technology access, which has been a limiting factor to SDV integration in many places (Boer et al. 2017, p. 21). Vehicles request relevant information about their current position from the cloud, overcoming the limitations of sensor-based information (Kumar et al. 2012). Both automotive and ICT companies have also had to invest heavily in their frequency communication infrastructure as there was an unwillingness by governments to finance these systems at the speed required to facilitate SDV integration.

*Political barriers* Since SDVs were first developed, there has been a difficulty in establishing standardisation between companies and countries. It has been challenging to develop protocols, with some claiming that regulation has been too stringent, halting progress, while others have stated that it has not been stringent enough. Governments have found it difficult to strike an appropriate balance between the two and there has also been a great deal of diversity with SDV policies globally, ranging from extremely detailed and dense (EU, US, and Japan) to non-existent (Eritrea, North Korea, and Somalia).

*Economic and geographic barriers* One barrier for SDVs adoption has been their cost and the infrastructure required to facilitate them. It has been costly to implement policies to accommodate SDVs, so SDVs have largely been adopted by wealthier countries. They have mostly remained untested in many of the world’s poorer countries, which is proving to be a key concern in global SDV and social justice circles. Even within richer nations, there has been a wide divergence in acceptance rates of SDVs. For example, willingness-to-pay studies have varied widely amongst nationalities, with many of these divergences remaining largely unchanged since 2017, despite national efforts: ‘Italian participants were most interested in using autonomous vehicles (65%), followed by the Spanish participants (54%), the French participants (51%), the Belgian participants (50%), the German participants (44%) and the American participants (32%)’ (Johnsen et al. 2017, p. 25). Location has played a fundamental role in the acceptance or rejection of SDVs, due to varying local attitudes, reliance on employment in driving professions, and technological capabilities, as well as economic stability of the country. For example, despite there being a greater acceptance rate among Italian and Spanish citizens, the economic instability of both regions over the past decade has inhibited the integration of SDVs.

*Employment barriers* One of the main inhibitors to the acceptance of SDVs has been a concern around job security. There has been an increased concern in recent years about SDVs replacing taxi drivers, bus drivers, delivery drivers, and anyone dependent on driving as a profession. Many trade unions and organised workforces in these areas have petitioned and protested at the replacement of workers in these sectors. Animosity towards SDVs from these groups has led to isolated incidences of abuse towards SDV taxi managers, destruction of vehicles and protests outside Waymo headquarters in Mountain View, California.

*Social barriers* There has been a lot of negative publicity around SDVs, particularly around fatalities they have caused, such as the Uber accident in 2018 (Levin and Wong 2018). There have been many cases of residents harassing SDV drivers, slashing tyres on vehicles, and throwing rocks at the SDVs (Cuthbertson 2018). The media has sometimes been criticised for focusing on many of the negative aspects of SDVs, such as the crashes and fatalities, which has affected public understanding and acceptance of the vehicles. Providing a level of trust amongst the public in relation to crashes, hacks and malfunctions has been one of the greatest challenges for SDVs market integration.

*Data protection and privacy barriers* Since the creation of the General Data Protection Regulation (GDPR) in the EU in 2018 and the many controversial data leaks and privacy debacles over the past 7 years, there has been a heightened concern about data protection and privacy, which has inhibited SDV deployment. SDV developers have been trying to navigate between privacy and data protection on the one side, and the need for vast amounts of processing data for SDVs to function, on the other. After the first large fine of €50 million against Google back in 2019 from European regulators (Porter 2019), which created a snowball of large ICTs being heavily fined, there has been a strong fear in the industry about breaching the GDPR. The GDPR has sometimes proven to be a hurdle for SDV manufacturers selling into the EU market, whereas countries not abiding by this regulation have been able to develop their SDV data-dependent algorithms quicker.

Because SDVs are relatively new to the market, it has also been difficult to estimate user acceptance. In many reports, there is an expressed fear that others will have access to your data. Some organisations have long established protocols to ensure that users’ privacy is protected when selling their SDVs (NADA 2018).

*Legal barriers* There has been a difficulty uniting cohesive legal analysis due to national differences on road traffic and transportation. One of these barriers has been determining accountability in cases of accidents. Some manufacturers have tried to keep accountability in the hands of the driver, keeping SDVs at level 3 automation. However, this has also prompted some manufacturers to take full responsibility in order to promote trust in their vehicles. The different levels of accountability have led to some confusion in the insurance industry about how to deal with accidents and how to define culpability.

## Ethical, Legal, Social and Economic Impacts of SDVs Between 2019 and 2025

### Ethical Impacts

*Safety and prevention of harm* Over the years, there has been a great deal of discussion about whether non-autonomous driving should be banned when we reach a level where SDVs can safely and easily replace non-autonomous driving. When SDVs become used so prevalently, it begs the question whether non-autonomous vehicles should be banned for safety reasons (Nyholm 2018, p. 6). Because the rollout of SDVs has been so slow, this has not been a pressing question, thus far, but the US and UK road safety authorities have indicated that this is an inevitability within the next 50 years. Meanwhile, groups such as Humans Against Autonomous Vehicles (HAAV) have strongly opposed SDVs because they are not safe enough to drive and are just “glorified smartphones”.

Since SDVs were first developed, the issue surrounding the vehicle’s decision-making in unavoidable crash situations has been widely discussed. There have been several ethical guidelines and best practice documents established to assist SDV programmers in developing ethically-sound crash algorithms. However, these guidelines have been criticised as being too vague, incoherent, and ineffective at addressing some of the most problematic issues with SDVs, namely, developing driving algorithms for crash scenarios. Very few people would buy an SDV if they prioritised the lives of others over the vehicle’s driver and passengers, but if they only aim to protect the driver, they may crash into children or light vehicles, instead of other cars, walls, or lampposts (Contissa et al. 2017, p. 67).

If safety is prioritised, they may swerve towards a motorcyclist wearing a helmet, as opposed to one without a helmet, because they would be more likely to survive in a crash (De Sio 2017, p. 425). If algorithms target those less at risk, then people may start to take unsafe activities in order to become safe, i.e. cycling without a helmet so that SDVs view you cautiously, thus avoiding collision (Johnsen et al. 2017, p. 42). SDV manufacturers have taken different approaches to these problems, with some attempting to constrain SDVs at level 4 automation to areas that prohibit non-autonomous vehicles, because the uncertainty of non-autonomous driving is one of the biggest risks to SDV driver safety. SDV manufacturers have been aware of the problematic issues with crash algorithms, but have rarely addressed this issue head-on, consistently stating that their SDVs will reduce crashes in the long-term, while implicitly conceding that there will be some degree of accidents and issues along the way.

*Moral algorithms* Algorithms determine statistical likelihoods that certain groups of people would be more likely to die in a collision (Nyholm and Smids 2016, p. 1285). Questionnaires and surveys meant to identify driving behaviour have been shown to be inaccurate because some people feel pressured to give more self-sacrificing, altruistic answers, rather than honest responses about how they would react in real-life situations. Critics have stated that it is naïve to assume that people are generally self-sacrificing in split-second decisions, which has been verified in repeated driving simulations and experiments for over a decade now (Sato et al. 2013). Therefore, creating crash algorithms based on social values, or even individual values, has been difficult to incorporate within SDV driving algorithms. While there have been guidelines and recommendations, regulation is still not fundamentally clear for SDV programmers, who still try to base the vehicles’ decisions on least-likely determinable harm done in a situation.

*Autonomy* The criticism that programmed responses remove control from the human being in driving circumstances has gained significant prominence in debates on SDVs. We lose the choice and ability to make our own decisions in the car’s navigation. 2021 witnessed much greater concern about individual autonomy in SDVs, as there were cases in China where the vehicle took control from the driver in non-automation mode. In cities where level 4 automation is in place, there have been personal accounts of individuals feeling a loss of control in these vehicles. In other instances, SDVs have been programmed to abide by speed limits and rules of the road, thus removing driving freedoms. In California recently, a pregnant woman went into labour and had to be rushed to hospital, but there were considerable delays because of the SDV’s speed limit regulation, which almost resulted in delayed birth injuries for the new-born.

*Responsibility* There has been a concern that SDVs are threatening our free will and moral responsibility, because of an overreliance on algorithms and artificial intelligence in SDVs (CNIL 2017, p. 26). There has been a worrisome trend towards shifting responsibility by autonomous vehicle owners, preferring to alleviate themselves from responsibility by driving in autonomous mode. During several interviews in recent years, many drivers said that they were not responsible for the actions of their SDV and subsequently should not be held accountable or liable for accidents involving their vehicle.

*Rights* Policymakers have identified that while SDVs open the possibility for more people to use them (such as the elderly, disabled, and blind), it also poses the challenge of who one does deny the right to use them. As of now, countries are still following non-autonomous driving policies in relation to driving capacity, as most still require level 3 automation, but once they reach widespread level 4 and 5 rollouts, more groups will benefit from them.

*Insurance and discrimination* Now that cars can retrieve a wide array of driving habits, patterns, and behaviours, it means that if insurance companies gain access to this information, insurance could be tailored to meet individuals’ driving performance. While being heralded as a positive move towards providing better insurance premiums to safer drivers, others have proposed that it would infringe on people’s sense of privacy, with the feeling of constantly being monitored in the vehicle. Others have disavowed it because of the imbalance in insurance between manual cars and SDVs—namely, that insurance companies will provide better conditions for SDV drivers who allow their data to be monitored by insurance companies, to the disadvantage of non-SDV drivers.

*Privacy* As a result of the large amounts of data retrieved from SDVs, policymakers have had to identify methods to ensure privacy and data security and if a SDV is breaking the law, whether the police should be allowed to hack it or not. Regulators have determined that strong levels of encryption, anonymization and aggregation need to be implemented to protect the individual’s personal data. Many automobile manufacturers are promoting their DRIC “data remains in car” compliant approach (CNIL 2018), which attempts to process data within the car, rather than being transmitted to different service providers or third-parties. This has been recommended since late 2024, but manufacturers have found it technically challenging to abide by.

### Legal Impacts

*Data and privacy* SDVs produce huge amounts of data and require large processing capabilities. The massive amounts of data required to operate SDVs have long raised issues about individuals’ privacy—if individuals are identifiable, who has access to this data, and what can be done with it (Gogoll and Müller 2017, p. 685). There has also been debate over whether data acquired from SDVs can be used as legal evidence; for example, if the driver was in control of the car at the time of an accident, could that evidence be used in court to determine liability (Johnsen et al. 2017, p. 53). So far, there have been several court cases in the US, Japan, and Australia to determine accountability of crashes involving level 3 vehicles. In most cases, the in-car cameras and steering-wheel sensors have demonstrated that the driver was indeed at fault. However, there were two cases in California that demonstrated that it was indeed a manufacturing flaw which caused those accidents.

There are global concerns about how long data should be stored; where it should be stored (e.g., on the car’s hard drive, the manufacturer’s cloud platform or an independent cloud platform); who should be granted access to this data; under what conditions; what happens to the owners data when they sell the car; how will the data be protected from being hacked; and who owns this data. So far, it has been difficult to have any international uniformity on these issues, except for EU-states, where there has been a much greater cohesion of legal frameworks on SDVs. This is expected to be a prime topic of concern in the third annual autonomous driving national leaders meeting later this year.

While driver and passenger data were the primary concern in 2024’s international meeting, external vehicle data will be a predominant topic this year. Sensors collect information about the environment, which threatens bystanders’ privacy. Because car companies are compiling mixed data (both personal and non-personal), it has been a little unclear how they are abiding by the GDPR and other regulations. In addition, they have also had to incorporate how they were securely and safely protecting privacy in accordance with ePrivacy Regulations (ePR) created to ensure that automotive companies abide by its guidelines. The European Automobile Manufacturers’ Association (ACEA) and the Council of the European Union have been paramount for ensuring that these governments implement the ePR and that those working in the industry follow the recommendations outlined (ACEA 2018).

*Cyber-security* People have been fearful that SDVs will be easily hacked because of the abundance of digital infrastructure required for them to work. Criminals have been making explicit use of the data that they retrieve, hacking the vehicle and getting it to perform actions the user is unaware of, unable to undo, and maliciously causing harm to the individual(s) in the car (Bowles 2018). If cyber-criminals take over a vehicle, they can cause minor nuisances, such as opening and closing windows, or they can create greater threats such as disabling the car’s functionality to read stop signs, maliciously causing vehicles to crash and harm its passengers, or using SDVs for terrorist purposes, such as transporting and detonating remote-controlled bombs. While there is a greater need for transparency from car manufacturers, there is the problem that cars will become more vulnerable as a result. So far, there have been only a few minor issues related to cyber-security, such as the case in London where attackers found weaknesses in SDVs through crypto malware and were able to extort money from the passengers before releasing control of the vehicle. However, these were isolated incidents and most of the cybersecurity insecurities have been identified by grey-hat hackers before malicious incidences occurred.

There has been a greater emphasis on strengthening counter-measures to avoid these situations. For example, in January 2025, UK police were granted the ability to take over cars that are hacked or under control for malicious purposes. This was done using Decentralised Environmental Notification Messages (DENM), which are messages exchanged between peer-to-peer SDVs and their digital infrastructures (Article 29 Data Protection Working Party 2017, p. 3). If there are abnormalities, DENM sends messages that indicate that the vehicle has been hacked to the police, through certification and Public Key Infrastructure (PKI) architecture (Article 29 Data Protection Working Party 2017, p. 4). However, these anomaly-based detection methods can identify a lot of attacks, but miss others, so there have been developments towards remote attestation methods, which check protocols before granting access to services (Kylänpää 2017). If there are abnormal issues addressed during this process that indicate potential hacking, this is relayed to the Police ICT Departments for further testing before intervention.

*Liability* At levels 0-2 automation, it has always been clear that, legally, the driver is responsible for the car’s behaviour. SDVs become a liability issue at levels 3 and 4, because of the uncertainty of who is responsible in cases of accidents. It is very important, under law, to identify who is responsible for the vehicle and under what circumstances. So far, some traditional insurance companies have established insurance policies for SDVs, with premiums at the same rate as non-autonomous vehicles, unless the driver grants them access to their SDV data. Since 2020, some of the main issues relating to SDV liability have been:

- Determining accident liability if the driver can concentrate on tasks other than driving. For example, if the car is in self-driving mode and the driver is reading, but needs to quickly take control of the wheel, and fails to do so in time, should the driver be held accountable? So far, manufacturers have largely claimed responsibility for crashes at level 4. However, at level 3, manufacturers have tried to place liability with the driver, but the nature of our cognitive setup disallows an exact and smooth transition between vehicle and driver in these situations. While drivers are not permitted to do other activities that would prevent them from taking control of the wheel during level 3 automation, the transition between the autonomous system and the human agent has been problematic to determine liability. There have many court cases that resulted with the driver facing diminished liability in these situations. There has been a greater push for manufacturers to progress to level 4 automation to overcome many of these issues.
- In incidents where it has been reported to be the fault of the vehicle, it has been difficult to identify when and where the reported malfunction on the vehicle occurred, making it a challenge to identify liability. There is an increased impetus to improve malfunction detection, and several manufacturers are implementing a range of on-board and external cameras and sensors to better determine time and origin of malfunction. However, this has been met with some hostility from passengers, worrying that they are losing their privacy and freedom within the vehicle. Manufacturers attest that this data will only be retrieved in cases of accidents and at the discretion and consent of the driver and passengers.
- There have been problems determining liability in situations where drivers activate the car when they should not have, or they do not take over control when requested. For example, there was one notable case in 2023 in Canberra, Australia, where a driver did not take over the wheel when prompted and was criticised for actively allowing the vehicle to crash to remove himself from potential liability. This has been a problem for SDV manufacturers and has led some companies to try to bypass level 3 automation to overcome this issue.
- Problems have arisen when the vehicle and driver have reacted at the same time, which has created problems for determining liability. For example, there was a situation in Seoul last year (May 6th, 2024), where the driver turned the steering wheel to the right to avoid a collision, but the SDV tried to veer left to avoid the collision. Both actions led the vehicle to crashing into the car in front. Luckily, nobody was badly injured, and the manufacturer admitted responsibility after reviewing the driver’s on-board footage because it was not clear that the vehicle should take control. Manufacturers have begun implementing audio and visual signals to demonstrate the car’s actions to minimise these types of incidents.
- In a Volvo SDV test-drive in December 2021, there was thick fog on the road, impeding the car’s object recognition sensors. A dog came onto the road, but the SDV’s was unable to detect it on time and the car skidded out of control into a bollard nearby. The driver would have seen the dog earlier, with the use of his high-beam fog lights and could have avoided the crash. SDV sensors has progressed immensely over the past few years, but manufacturers still state that there will be occasional glitches in difficult driving conditions, which has forced companies to limit automation in difficult driving conditions, such as heavy fog and snow.
- It is difficult to determine if the driver is liable in situations where their SDV breaks the law, if the driver is not required to monitor the vehicle’s actions. So far, in the locations where level 4 vehicles have been integrated, manufacturers state that they are strictly following local laws and rules of the road, and this issue has not yet materialised.

### Social Impacts

*Joy of driving* For many, SDVs threaten to take away one of the primary pleasures of vehicles—the joy of driving itself (Kemp 2018). While for some driving is a necessary ordeal that must be endured, for others, it is a form of pleasure in itself: a sense of control, a form of relaxation, a sense of adventure, and a connectedness with their surroundings, that is being threatened by SDVs. 2022 and 2023 saw a rapid increase in the number of driving enthusiast affiliations attempting to ensure SDV safety does not force their non-autonomous vehicles off the road. There is a conflict between those who promote the reduced numbers of traffic deaths and those who want to protect their right to drive.

*Gender differences* Many years ago, the BRAVE Project was one of the first to highlight that there are different perceptions about SDVs between men and women. Men have had less worry about embracing SDVs, while women have been less enthusiastic and more fearful about their safety (Johnsen et al. 2017, p. 27). Men have been buying SDVs at a greater rate than women, with an approximate 60–40 split in SDV usage. Manufacturers are investing in female-focused SDV advertising.

*Inclusion* SDVs hold the potential to reduce inequalities and promote inclusion amongst drivers by allowing certain groups (senior citizens, non-drivers, and people with disabilities) access to automobiles that were limited, or unable to, previously (Johnsen et al. 2017, p. 56). However, because of our current low levels of automation, this has yet to materialise, although many of these groups have indirectly benefitted from the use of SDV ride-hailing.

*Car-sharing* While SDV car-sharing has not yet materialised because of low levels of automation, they hold the possibility of changing the nature of car ownership in the future. Some propose that SDVs will not remain unused in garages or parking lots but will be shared amongst groups of people and used throughout the day, when we get to widespread level 4 and 5 automation (Johnsen et al. 2017, p. 55). Google’s Waymo has been pioneering SDVs ride-hailing as far back as 2018 and have since introduced preliminary pilots in several cities throughout the US (Griswold 2018). There were a few incidents in 2023, where passengers were not allowed to leave the car because of a glitch in the payment system, but overall, SDV ride-hailing has been a huge success and is set to expand globally.

*Travel behaviour and demands* It is still unclear if total travel miles increase as a result of improved comfort, ease of travel, and the ability to multitask while in the vehicle. So far, the limited integration of SDVs indicates that people travel more often as SDV use reduces many of the stresses found in traditional driving. In addition, fuel costs have been decreasing in five of the seven cities where level 4 automation has been implemented, because of more efficient driving, while the other two cities showed no change. In the past, it was assumed that insurance costs for SDVs would decline with a lower number of accidents. However, insurance companies are still dubious about the safety of SDVs and have kept insurance costs mostly the same as for non-autonomous vehicles, unless drivers can prove their safe driving through their SDV data. While SDVs initially had a higher number of accidents per mile than traditional cars, this was simply because they were in such early stages of development.

*Decreased urbanisation* What has been happening in some cities is that people are beginning to rent further away from the city centres because of the ease of commuting and reduced costs of running their SDV. There is less of a need to live in cities, which have started to see a reduction in urbanisation, allowing for a more evenly spread out population throughout the region. It has started to take some of the strain off amenities and busyness of very congested cities, while also potentially eliminating the need for so many parking spaces in the future, when SDVs can be used throughout the day (Lubell 2016).

*Environmental* There is an uncertainty about whether SDVs are ameliorating or exacerbating congestion levels. So far, people with SDVs have increased their overall travel time because they see it as less of a burden. Early signs indicate that increased efficiency of SDVs is reducing carbon emissions more than non-autonomous vehicles. SDV developers have been trying to walk the tightrope between ensuring their vehicles are environmentally-sustainable and having economically-affordable vehicles. Some manufacturers have placed a greater emphasis on emission reductions with the foresight that governments are implementing harsher penalties for poorly performing vehicles.

### Economic Impacts

*Job-losses* In the past, there were concerns that SDVs would lead to job losses for ‘taxi drivers, parking attendants, valet parkers, car mechanics, meter attendants, traffic officers, and potentially bus and freight drivers’ (Lari et al. 2015, p. 758). There was also the issue that there were not enough people to drive trucks in places such as Canada (CBC News 2018), so truck manufacturers, such as Mercedes, noticed this trend and capitalised on autonomous trucks (Mercedes-Benz 2018). They have been testing level 5 trucks in locations where it is too dangerous or unsuitable for humans to drive, since Autumn 2023. Uber also saw that many of its drivers could become unemployed because of SDVs, so they have created computer science, engineering, and maintenance programmes for those interested in upskilling and transitioning professions (Engelbert 2017).

*Competition* As a result of the large investments and technological capacities of SDV development, we have seen several smaller automotive companies beginning to dissolve because they will be unable to compete with these giants going forward. While SDV start-ups flourished in the early infancy stage, the larger players have started outcompeting them with innovation, thus minimising the competitive market of SDV manufacturers.

*Luxury vehicle business* Some of the luxury vehicle manufacturers were worried about how SDVs would impact their business models, especially if driving were solely relegated to a hobby. However, some manufacturers have flourished through this period, with Audi and Mercedes taking leading roles in the SDV market (Autotech 2018). However, companies such as Ferrari, Lamborghini and Lexus are trying to re-market their vehicles and are increasing investment into their ‘drive for fun’ initiatives and racing tracks.

*Digital divide* SDVs are very expensive, which has limited ownership to rich people (Oliver et al. 2018). It is difficult for poor people to drive SDVs and may become problematic when it becomes the prevalent form of transportation. There are concerns that the increased safety of SDVs may cause non-SDVs to be unsafe and eventually prohibited from being sold, limiting people to more expensive SDVs.

Cost reduction: In the past, it was suggested that SDVs would cause insurance and energy costs to decrease, but we have only witnessed minor changes. While SDVs are hailed as safer, which should have reduced insurance costs, this has still not materialised in practice—insurance costs on SDVs have not become cheaper than non-automated vehicles.

*Road infrastructure* There has been a lot of debate over whether governments should maintain existing infrastructure or start implementing a more digitised infrastructure to accommodate for SDVs (Peters 2017). So far, SDVs have had to develop to understand human signs, rather than digital signs. Furthermore, there has been a public outcry about governmental investment in SDV infrastructure, with many claiming that it should be partly funded by auto companies. In late 2024, demonstrations in France and Germany called on SDV manufacturers to help cities pay for SDV infrastructure in the coming years.

*Law enforcement income* There has been a concern, in London and Mountain View, California, that SDVs will impact income generation of law enforcement. With more law-abiding vehicles, there has been a marginal and slow reduction in speeding and illegal parking. While more law-abiding vehicles is obviously a good thing, it still means a lost form of revenue generation by the police (Marshall and Davies 2018).

*Electricity and power* While SDVs have been powered by a mix of electric and traditional fossil fuel, there has been a strong push by many governments to switch to all electric. For example, the UK government stated back in 2018 that more than half of all vehicles on the road should be electric by 2030 (Harrabin 2018). While this is nowhere near achievable in the next 5 years, electric SDV use is being hailed as the way forward to reduce our automobile dependency on fossil fuels.

## Mitigating Negative and Accentuating Positive Impacts of SDVs Between 2019 and 2025

As far back as 2019, there have been many different actions to mitigate negative impacts, while accentuating the positive impacts, of SDV technology, through national, international and supranational legislation and policy. One of the ways this was achieved was through national standardisation protocols between policy-makers, auto manufacturers, computer scientists, and transportation agencies. Standardisations have been created to ensure sufficient cyber security capabilities for SDVs are developed and implemented; minimum requirements established for the use of sensor technology; safety levels have been incorporated into earlier vehicle regulations to include hardware standardisations; and there have been several layers of enforced testing for different levels of vehicle automation.

National governments have implemented an array of different measurements and regulation to ensure that safety standards are being met. Many countries have heavily invested in their own independent testing, as there were several concerns related to scientific bias in manufacturing testing. In doing so, the US, Canada and Japan have created a greater transparency around SDV regulation. In total, 65 countries have developed their own SDV driving tests and licensing laws, while also enforcing safety regulations for manufacturers to demonstrate that these vehicles are safe to drive prior to being sold.

There have also been strengthened measures to inform the public about SDVs, how they function, and how non-autonomous drivers should interact with them on the road. This has led to a greater public trust, in conjunction with a large increase in media public awareness campaigns from car manufacturers. There has been a greater emphasis placed on the benefits retrieved from the big data of SDVs, but strict procedures and guidelines have been instituted to ensure personal data is anonymised and encrypted in accordance with GDPR, which has been a milestone for privacy protection over the past 7 years.

The automobile industry has had to adapt its earlier approach to the design process of their vehicles, with a greater emphasis on responsible innovation and value-sensitive design. The increase in ethical evaluations of SDVs resulted from state-supported initiatives and the establishment of oversight bodies, such as the UK’s Centre for Data Ethics and Innovation, and Singapore’s AI Ethics Council. Manufacturers have also had to increase transparency, while also providing guarantees for the life-span of their vehicles. Free software upgrades are mandatory for a 5-year period with all SDVs sold in the US, Canada, the EU, the UK, China, South Korea and Japan.

Where software updates occur on a regular basis, manufacturers have provided extensive guidelines about these requirements. SDVs have a built-in locking system that will prohibit drivers from using the cars unless their systems are updated. The cars also have clear and purpose-driven maintenance notification for drivers. Depending upon the seriousness of the maintenance, vehicles may prohibit drivers from operating. There has also been collaboration and agreement through the SDV Fair Use Initiative (SDVFUI) to ensure fair sharing of intellectual property for increasing safety in vehicles.

Since 2023, it has been evident that incorporating more digital infrastructure on our roads would be beneficial for the successful implementation of SDVs. While we are still in early stages, SDVs could be used more optimally with improved digital and physical infrastructure. Civil society organisations have been decrying the possibility that all citizens will have to pay extra for those making the change to autonomous driving, when they are not the ones benefiting from them. Policymakers have been negotiating with SDV manufacturers and owners about paying higher taxes to fund the infrastructure required to accommodate SDVs.

## Steps Towards a Desired Future and Avoidance of an Undesired Future

This scenario has outlined many different issues, risks, and possibilities of SDVs in the year 2025. It is very important to reflect on some of the situations found in the scenario, and highlight those that are desirable by 2025, those to be avoided, and how to go about doing this. National, international and supranational institutions should be responsible for ensuring that citizens are protected from the over-eagerness of manufacturers to put their vehicles on the road. What became evident in the development of this scenario is that some of the main facilitators for SDV manufacturing will come from market, economic, and efficiency incentives (facilitators section); this should not jeopardise the safety, security, and employment of citizens (inhibitors section). Therefore, the SDV industry needs to be well regulated to ensure the safety of their vehicles through the effective implementation of SDV regulatory institutions.

In the privacy sections (in terms of both ethical and legal impacts), it was shown that under regulated SDV development has the potential to have negative ethical impacts on the privacy of drivers, passengers, pedestrians, and road-users, while also having legal implications in terms of data protection and privacy law. These sections demonstrate that there needs to be adherence to current regulations for the effective control of data generated, retrieved and used by SDVs. Clear delineations need to be established about what constitutes *essential data* for the vehicle’s mobility and if this contains personal and private information. There needs to be clear indication that if essential data contains personal or private information, then it should be strongly anonymized, aggregated, and secured, to protect the privacy of drivers, passengers, and other road users. If it is non-essential data, then there should be adequate policies to ensure that they are not retrieved or stored as a result of using an SDV, unless explicit and informed consent is given.

As was made clear in the legal impacts section of this scenario, European governments need to effectively integrate the tenets of the GDPR into the automotive industry to effectively assure citizens that their personal data will be protected if they use SDVs. Automobile manufacturers have the responsibility of identifying the purposes for which the car collects data in order to demonstrate their compliance with data protection law. For instance, there needs to be careful analysis if this data will be used for advertising, customised pricing, or to sell additional products to the car owner, and either ensuring the owner is aware of these, and consent to it, or prohibit use of data in this way, altogether.

As also brought out in the scenario, there is a current legal concern that SDVs will be used for malicious, illegal, and fraudulent purposes. SDVs may threaten the safety and security of passengers, pedestrians, and cities if controlled or hacked my criminals or terrorists. Therefore, data collected within the vehicle may become important for law enforcement officials in situations where SDVs are hacked or used for malicious purposes. Police authorities should be allowed to identify illegal SDV activity, as long as it does not infringe upon the privacy of innocent citizens (cybersecurity section). Methods such as DENM, certifications, cryptographic signatures, and attestation methods require heavy investment by automotive companies and need to be fit for purpose; which illustrates the tensions discussed in the paper between safety and economic concerns. There needs to be careful statutory regulation, third-party testing, and planning for the security of these technologies, which may inhibit the speedy deployment of SDVs (inhibitor), but which will ensure greater security of these vehicles (facilitator).

One of the main social and economic issues imagined for the future will be the challenge of transitioning between non-autonomous vehicles and autonomous vehicles. A key foreseeable issue will be the adequate implementation of digital infrastructure to accommodate SDVs. Therefore, policymakers will need to ensure that there is a smooth transition between traditional infrastructure and the digital infrastructure of the future. For the foreseeable future, SDVs will have to use our current road signs lights and markings to navigate on roads. However, these may eventually be replaced by ‘digital infrastructure’. While this is not likely to transpire by 2025, governments and companies should already be preparing for this transition as it will be one of the most costly and time-consuming processes in the materialisation of widespread SDVs usage.

While it was imagined that SDVs will offer the potential to reduce our ecological impact, reduce traffic and congestion, and improve driving-times (social drivers), there is nevertheless also the possibility that they will be used more often because of ease of use (social impacts). Therefore, it makes sense that policymakers would need to take careful steps to ensure that they are not ‘overused’, thus reversing many of their social and environmental benefits. Policymakers may be able to do this is through greater investment into SDV public transportation systems to ensure convenience, cost and energy reductions. This may also have the positive social impact of preventing poorer citizens from being excluded from the transportation system—the digital divide issue, highlighted in the economic impacts section of this scenario. Furthermore, careful attention must be placed on ensuring more inclusionary SDVs, especially when they reach level 4 and 5, especially the elderly, handicapped, and those who cannot drive, can be granted accessibility to SDVs, in an attempt to reduce many rights-based issues when it comes to automobile usage, as discussed in the ethics section of this paper.

## Conclusion

SDVs are set to dramatically transform the way we live in the future, but it is often not abundantly clear *when* in the future these transformations will happen, or, relatedly, the future is conceived as being too distant to have significant enough urgency for policy considerations and implementation now. This paper presents the policy-focused scenario approach which was developed as one way to evaluate these emerging concerns. Scenarios are useful for planning and mapping the potential trajectory of emerging technologies and provide us with insightful visions of what the future may hold and how policymakers can either put steps in place for a desirable future. The approach we report on in this paper demonstrates a variety of inhibitors and facilitators of SDV development by the year 2025, indicating concerns and methods for policymakers to take into account in the coming years. The scenario also highlighted many of the most current and widely discussed as well as likely ethical, legal, social and economic impacts of SDVs in the coming years and steps and procedures that should be taken to reap the benefits, while curbing the unwanted outcomes, of these emerging technologies. While scenario planning is not an exact science, it provides policymakers with additional insights of how their actions now will affect the future development and use of emerging technologies, such as SDVs.

## Footnotes

1 SHERPA is a European Commission-funded project in the Horizon 2020 programme.  In collaboration with a broad range of stakeholders, the SHERPA project will investigate, analyse and synthesise our understanding of the ways in which smart information systems (SIS; the combination of artificial intelligence and big data analytics) impact ethics and human rights issues. It will develop novel ways of understanding and addressing SIS challenges, evaluate with stakeholders, and advocate the most desirable and sustainable solutions. SHERPA has a budget of € 3.0 million and a running time of 3 ½ years (May 1, 2018–November 1, 2022). It is carried by a consortium of eleven organizations from the European Union and supported by an extensive stakeholder advisory board that includes many major and international organisations with an interest in AI and Big Data. See our website: https://www.project-sherpa.eu/.

2 While grounding the workshop on technical aspects of SDV development was important, during the iteration stages, everyone agreed that it was somewhat irrelevant and detracted in the writing of the scenario.

3 During the workshop stage, most of the input from the stakeholders was a collective effort, so the thoughts and viewpoints were often a collaborative effort, rather than specific individuals’ perspectives being singled out.

4 In-text referencing was used because it was the required referencing system for Science and Engineering Ethics, but also because it lends clear scientific credibility of the document to policymakers.

5 The Waymo Centauri b was named after the closest habitable exoplanet in the solar system.

6 However, it must be made clear that many of these issues are interrelated with one another. For example, there is a close link between the issue of responsibility in ethics and accountability in legal, similarly with the issue of privacy.
